# Fundamentals of Global Modeling for Polymer Extrusion

**DOI:** 10.3390/polym11122106

**Published:** 2019-12-15

**Authors:** Krzysztof Wilczyński, Andrzej Nastaj, Adrian Lewandowski, Krzysztof J. Wilczyński, Kamila Buziak

**Affiliations:** 1 Polymer Processing Department, Faculty of Production Engineering, Warsaw University of Technology, 02-524 Warsaw, Poland; a.nastaj@wip.pw.edu.pl (A.N.); a.lewandowski@wip.pw.edu.pl (A.L.); kami.buziak@gmail.com (K.B.); 2 Politech Ltd., 86-031 Bydgoszcz, Poland; wilczynski_k@wp.pl

**Keywords:** polymers, extrusion, modeling

## Abstract

A review paper is presented on modeling for polymer extrusion for both single screw and twin-screw extrusion. An issue of global modeling is discussed, which includes modeling for solid conveying, melting, melt flow, and co-operation of the screw/die system. The classical approach to global modeling of the extrusion process, which is based on separate models for each section of the screw, i.e., solid transport section, melting and pre-melting sections, and the melt flow section is presented. In this case, the global model consists of the elementary models. A novel continuous concept of global modeling based on CFD (Computational Fluids Dynamics) computations is also presented, and a concept of using the DEM (Discrete Element Method) computation coupled with CFD computations is discussed.

## 1. Introduction

**Figure 1:**
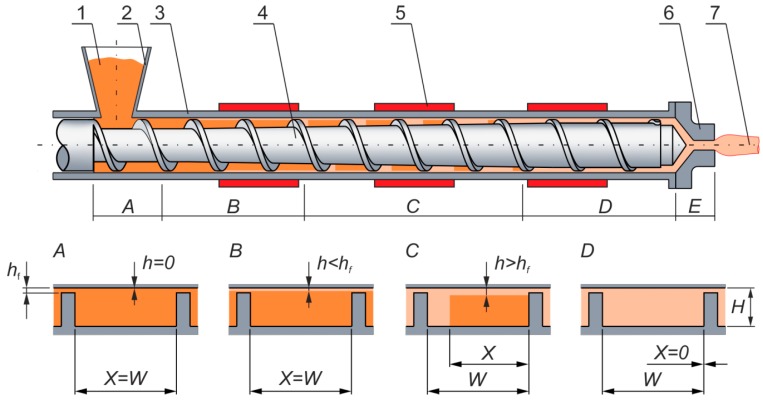
Scheme of the extrusion process: 1—solid polymer, 2—hopper, 3—barrel, 4—screw, 5—heaters, 6—die, 7—extrudate, A—solid conveying zone, B—pre-melting zone (delay zone), C—melting zone, D—melt conveying zone, E—melt flow zone in the die, X—width of the solid bed, W—width of the screw channel, H—height of the screw channel, h_f_—clearance between the screw flights and the barrel, and h—polymer melt thickness.

Extrusion (Figure 1) is the most important and most massive technology in the polymer processing industry. It is widely used for the production of film, sheet, pipe, and profiles, as well as for specialty processing operations, such as compounding, mixing, granulating, chemical reactions, and more. These operations are applied for most polymeric materials, and extrusion is mainly used for these operations.

There are several important functions of extruding machines, polymer transport (from a hopper to a die), polymer melting, pressure generation, material mixing for thermomechanical and chemical homogenization, and, lastly, product forming. Melting should be quick to provide enough room for good material mixing. Melting and mixing are fundamental in polymer processing and crucial for the development of novel, advanced materials, polymer composites, or polymer blends, as well as for material recycling of plastics.

Single screw extruders are generally poor mixers and melting progresses slowly. In twin-screw extrusion, melting progresses faster and mixing action is considerably improved. In order to improve melting and mixing actions, various screw configurations are applied, using elements that intensify these actions, e.g., Maillefer, Barr, Maddock, and many others.

The design of polymer processing is currently supported by computer simulations based on the mathematical models of manufacturing processes. Modeling makes it possible to predict the course of these processes on the basis of process data (material, operating, and geometry).

Several fundamental books or book chapters have been devoted to an extrusion process, e.g., by McKelvey [1], Tadmor and Klein [2], White [3], White and Potente [4], Tadmor and Gogos [5], White and Kim [6], Potente et al. [7], Rauwendaal [8], Campbell and Spalding [9], Chung [10], Agassant et al. [11], Manas-Zloczower et al. [12], Osswald and Hernandez-Ortiz [13], and many others [14,15,16,17,18,19,20,21,22,23,24,25,26,27,28,29,30,31,32,33]. However, the subject of global modeling and the continuum approach to this was not considered. Some of these books also contain an excellent literature review, e.g., by White and Potente [4], Rauwendaal [8], and Agassant et al. [11].

## 2. Modeling of the Extrusion Process

It is a particularly important challenge where the modeling of the two most important technologies are extrusion and injection molding. In these, there is a need for global modeling, i.e., description of the solid transport, polymer melting, and the melt flow. Polymer melt flows are relatively well understood. However, the transport of solid material as well as melting of the polymer are still poorly understood. The right polymer melting model is the basis for developing a comprehensive (global) model of the process.

### 2.1. Solid Conveying Section

Solid conveying in the single screw extruder was described first by Darnell and Mol [34] who elaborated on the model for material transport and pressure development. In this model, the solid particles are assumed to be rapidly compacted, which forms a non-deformable solid bed. This solid bed flows due to frictional forces exerted by the barrel and screw surfaces on the solid polymer granules. It is assumed that the internal coefficient of friction (polymer/polymer) is larger than the external coefficient of friction (polymer/metal). 

This fundamental approach was later extended by other researchers. Schneider [35] introduced anisotropy coefficients justifying that the pressure is not distributed equally in bulk materials. Tadmor et al. [2,36,37] introduced an energy balance considering the heat conduction into the solid bed. Campbell and Dontula [38] as well as Hyun and Spalding [39] defined an angle for the solid bed pressing force, which was not normal to the active flight of the screw. Chung [40,41] presented a different approach assuming that the solid bed moves because of the presence of molten polymer films at the metal surfaces. Essentially, the solid polymer is coated by a thin layer of polymer melt. In addition, several studies were performed in which the material data for the solid conveying section were no longer assumed to be constant, e.g., by Spalding and Hyun [42]. The research on solid conveying was recently reviewed in detail by Schöppner et al. [43,44].

Although numerous researchers extended the work of Darnell and Mol, the basic analysis remained relatively unchanged and was the basis for modeling the extrusion process.

All the models of solid conveying presented so far were based on the assumption that the polymer granules are moving as a solid bed without relative movement of polymer granules. However, some investigations, e.g., performed by Fang et al. [45] showed that this assumption cannot be valid any longer since relative movement of individual granules occurs in the screw channel. 

To solve the problem of modeling the solids’ transport within a single screw extruder, the discrete element method (DEM) was proposed, which is well known within the field of granular mechanics. The first research using this method in the field of solid conveying the extrusion process was performed by Potente and Pohl [46] who studied the hopper inflow behavior in single screw extruders. 

The fundamental studies on modeling the solid conveying in the single screw extruder with the use of the discrete element method were performed by Moysey and Thompson [47,48] who demonstrated the suitability of this 3D DEM approach for simulating single screw extruders. They presented the procedures for determining the coefficient of restitution [49], which is used as a material parameter in modeling, and performed the first simulations for compacting granules and the pressure/throughput relation in the feed section of the single screw extruder [50].

A further description of the solid conveying section based on the discrete element method (DEM) DEM was performed by Schöppner et al. [43,44] who developed the model, which fully comprehends the effects occurring in the solid conveying section and enables the calculation of a solid conveying zone with consideration of the pressure build-up and filling degree.

The discrete element method (DEM) is a very useful and powerful tool for modeling the solid conveying section of single screw extruders. However, it has two substantial drawbacks. It is time consuming and the material parameters, i.e., the coefficient of friction (CoF) and coefficient of restitution (CoR), are difficult to determine. Since it is time consuming, this method cannot be used for global modeling of the extrusion process, which requires hundreds of computing iterations.

Solid conveying in twin-screw extruders was studied, mainly for co-rotating extrusion, e.g., by Carrot et al. [51], Bawiskar and White [52], Potente et al. [53], and Wong at al. [54]. Studies of the solid conveying in counter-rotating extruders were relatively few. Doboczky first [55] discussed some of the problems of a solid conveying region, while Wilczyński and White [56] experimentally investigated it. Twin-screw extrusion systems are generally starved fed, and the solid conveying has less impact on global modeling than in flood fed single screw extruders.

### 2.2. Melting Section

**Figure 2:**
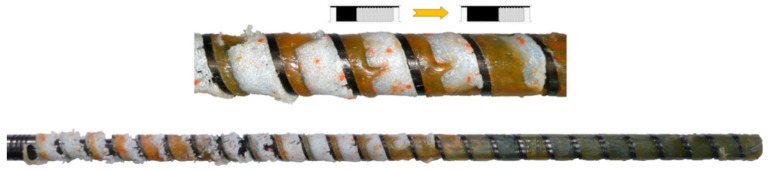
CSM melting mechanism (Contiguous Solid Melting) observed for flood fed single screw extrusion of polypropylene [59].

The first melting tests in the single screw extruder were performed by Maddock and Street [57,58] who used the ”screw pulling out technique,” which involves stopping the screw, rapidly cooling the machine, and then pulling out the screw of the machine. An analysis of the cross-sections of the polymer removed from the screw allowed us to get to know the melting mechanism (contiguous solid melting). According to this mechanism, a melt layer is formed between the hot barrel and the solid polymer, which is scrapped off by the transverse flow in the screw, and accumulates at the active flight of the screw. The solid is gradually decreased by the effects of heat conducted from the hot barrel and viscous dissipation within the melt (Figure 2) [59].

The experiments showed that two steps of melting can be distinguished, including the delay section (or pre-melting), which corresponds to forming and growing the polymer melt layer, and the melting section (or plasticating), which corresponds to the accumulation of the molten polymer at the active flight of the screw.

Several years later, Tadmor et al. [60,61,62] completed a similar experimentation and developed the first melting model for single screw extrusion, which was a crucial contribution to the theory of extrusion and allowed us to formulate the first computer extrusion model EXTRUD [63]. This fundamental melting model was built by determining the velocity and temperature profiles in the melt film and temperature profile in the solid bed (Figure 2). Then, an energy balance at the interface melt/solid and the mass balance in the melt film and the solid were performed, which allowed us to predict the melting rate. Later, these studies were extended, and more detailed models were developed for a solid conveyance [36,37] and for the delay zone [64]. Then, the basic extrusion model was improved.

Many researchers repeated the “screw pulling out” experiments, and, in the majority of cases, they confirmed the Tadmor melting model. However, there were some exceptions, especially in the case of melting PVC, which were reported by Menges and Klenk [65] and Mennig [66] who observed the location of the melt pool at the passive flight of the screw, which is opposite than in the classical model where the melt pool is located at the active flight (Figure 2). Dekker [67] observed that, in the case of PP, the solid bed is surrounded by the polymer melt and did not detect a melt pool on any side of it.

The fundamental model of Tadmor was later extended by other researchers. Donovan [68] relaxed the assumption of the constant velocity of a solid bed and introduced some acceleration parameters. Edmondson and Fenner [69] as well as Cox and Fenner [70] proposed a model which allows the solid bed to accelerate naturally, and allows for the presence of a melt film between the bed and the screw. Lindt et al. [71,72] assumed that the rigid solid bed is suspended in the melt, which was later improved by Elbirli et al. [73,74] by considering the solid bed deformation, and allowing for transverse flow of the melt around the solid. Pearson et al. [75,76] generalized this approach by formulating the most elaborate extension of the Tadmor model in the 5-zone model in which the solid bed, melt pool, and the melt films are analyzed separately. In addition, the melt film thickness on the barrel varies. Analytical melting models were also developed. The models of Vermeulen et al. [77], Pearson [78], and Mount et al. [79] analyzed the melting on the barrel surface. Potente [80] included the melting on the screw surface into his model. 

Housz and Meijer [81,82] pioneered the modeling of melting in multiflighted screws by modifying the classical Tadmor model. Elbirli et al. [83] as well as Amellal and Elbirli [84] developed non-Newtonian non-isothermal models that assumed the coexistence of fourth regions in a screw channel: solid bed, melt conveying, melt film at the inner barrel surface, and melt film surrounding the solid bed. Han et al. [85] considered the presence of six regions: solid bed, melt conveying, and four melt films at the inner barrel surface, screw root, barrier gap, and screw flights, respectively. Rauwendaal [86] has evaluated the performance of various barrier designs.

These studies presumed that the beginning of the barrier section coincides with the onset of melting, and the melting rate follows the rate of change of the cross-channel areas for solids or melt, which is not consistent with experiments. Gaspar-Cunha and Covas [87,88] developed a melting model where the onset and rate of melting are decoupled from the start and the cross-channel location of the barrier, and inserted this model into a global package describing flow and heat transfer along the extruder from the hopper to the die exit.

Single screw extruders may have barrels with a grooved feed zone and a grooved melting zone. Numerous experiments were performed to study the effect of the grooved barrel on extrusion performance, e.g., by Grünschloß [89,90], Chung [91], and Jin et al. [92]. It was validated that this system shows more efficient melting, higher specific throughput, uniform pressure buildup, and lower processing costs compared to other high-performance extruders. Avila-Alfaro et al. [93,94] presented the mathematical model for melting in the grooved plasticating unit, which was a revised version of the classic model of Tadmor with some improvements made by Vermeulen et al. [77] and Pearson [78].

The “screw pulling out technique” is a relatively time consuming and expensive method of studying melting in extruders. Therefore, other approaches were applied for observing the melting behavior directly in the extrusion process. For example, Zhu et al. [95,96] used glass windows in the barrel. Noriega et al. [97] applied advanced optical methods for visualizing the melting profile, and Wang and Min [98] used an ultrasound-based system for monitoring the polymer melting in a twin-screw extruder. Aigner et al. [99] developed a non-invasive ultrasonic measurement system for determining the melting behavior in a single screw plasticating unit. Very recently, Yu et al. [100] presented a visualization technique with a global transparent barrel equipped with four cameras to capture the flow patterns of a viscoelastic fluid in a novel type of co-rotating non-twin-screw geometry.

Similarly, as in the case of Darnell and Mol’s work for solid conveying, even though numerous researchers extended the work of Tadmor and Klein, the basic analysis remained relatively unchanged and was usually the basis for modeling the extrusion process. However, the models of the type presented so far are based on a prior assumed melting mechanism, which are not universal, and cannot be valid for all polymers, various operating conditions, and various screw configurations. These models can be useful only in qualitatively predicting the trends in melting polymers in single screw extruders.

Instead of the melting mechanism previously assumed, melting of a polymer in single screw extruders can be simulated by solving the conservation equations of mass, motion, and energy along with a constitutive equation for the polymer being used.

This different approach was proposed first by Viriyayuthakorn and Kassahun [101] who developed a three-dimensional FEM model without assuming any particular melting mechanism. The problem of the phase change was solved by using a functional dependence of the specific heat on temperature. The solution of equations of motion and energy provided the solid/melt distribution, which was defined by the temperature distribution. Syrjala [102] performed a two-dimensional attempt for simulating melting without any melting mechanism assumed. However, in both cases, the simulations were not verified experimentally.

This novel approach based on solving the conservation equations without an assumed melting mechanism seems to be very promising for the future work on modeling the melting, even though it requires very large computational capabilities. 

Altinkaynak et al. [103] performed intensive experimental and theoretical studies on modeling melting using this approach. The two-phase solid/melt flow was considered with the Cross-WLF model, which allows us to define a solid material as a high-viscous fluid, while a molten material allows us to define the material as a low-viscous fluid. Hopmann et al. [104] solved the equations of motion and energy using the finite volume method FVM with the Carreau model. Recently, Kazmer et al. [105] applied this approach to modeling melting in barrier screws, and Lewandowski and Wilczyński applied this approach to modeling in conventional screws [106].

Contrary to melting in single screw extruders, the studies on melting in twin-screw extruders were much more limited. These studies involved mainly modular self-wiping co-rotating twin-screw extruders, both experimentally (Bawiskar and White [52], Todd [107], Sakai [108], and Gogos [109,110,111]) and theoretically. Potente and Melish [112] as well as Bawiskar and White [113] proposed the models principally based on the classical Tadmor model [2] for a single screw extrusion, while assuming the progressive development of a molten layer from the barrel toward the screw root. Bawiskar and White [113] considered the formation of two stratified layers of melt in contact with the hot barrel and solid pellets in contact with the relatively colder screw. Potente and Melisch [112] proposed a model based on the melting of particles uniformly suspended in the polymer melt. A similar approach was presented by Liu et al. [95]. Vergnes et al. [114,115] and Zhu et al. [116] proposed the dispersive models based on the analysis of flow of the solid/liquid mixture with an equivalent viscosity.

**Figure 3:**
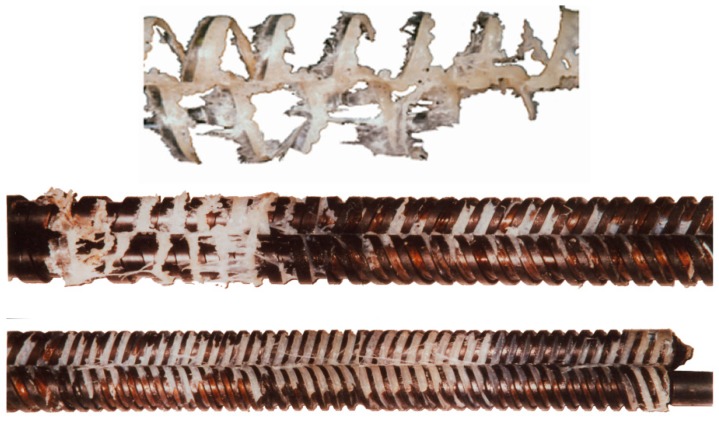
Melting mechanism for counter-rotating twin-screw extrusion [120].

Melting in counter-rotating twin-screw extruders is much less understood. Only limited observations were presented by Janssen [117]. White et al. [118,119] indicated that melting occurs much more rapidly than in intermeshing co-rotating twin-screw extruders. Wilczyński and White [56] revealed the mechanism of melting in intermeshing counter-rotating twin-screw extruders. According to this mechanism, melting is initiated both between the screws and at the barrel. The melting action between the screws is initiated by frictional work on the pellets by the calendering stresses between the screws. The melting action at the barrel is induced by a barrel temperature higher than the melting point and propagated by viscous dissipation heating of the melt film produced. Based on these observations (Figure 3), the models were developed for melting in both those regions [120]. Further studies of melting were reported by Wang and Min [98,121] and by Wilczyński et al. [122].

**Figure 4:**
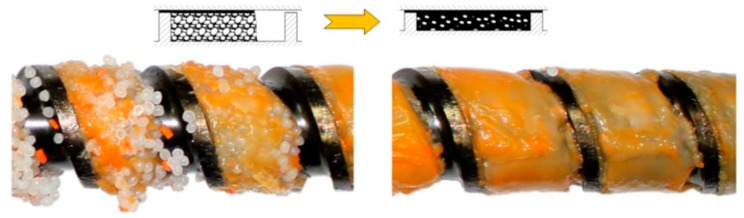
Melting mechanism for starve fed extrusion of polypropylene [59].

Although the flood fed single screw extrusion was extensively investigated and modeled, little information was presented on the starve fed single-screw extrusion. Several basic studies were performed by Lopez-Latorre and McKelvey [123], Isherwood et al. [124], Strand et al. [125], and Thompson et al. [126]. Recently, Wilczyński et al. [59,127] based on the experimental studies proposed the melting mechanism and melting model for the starve fed single-screw extrusion, and then developed the first computer model of this process SSEM-Starve [128]. According to this melting model, two stages of melting are distinguished. In the partially-filled region of the screw, the polymer granules are collected at the active flight and are generally melted by conduction. In the fully filled region, the unmolten solid particles are suspended in the previously molten material, and melting progresses through heat dissipation (Figure 4).

**Figure 5:**
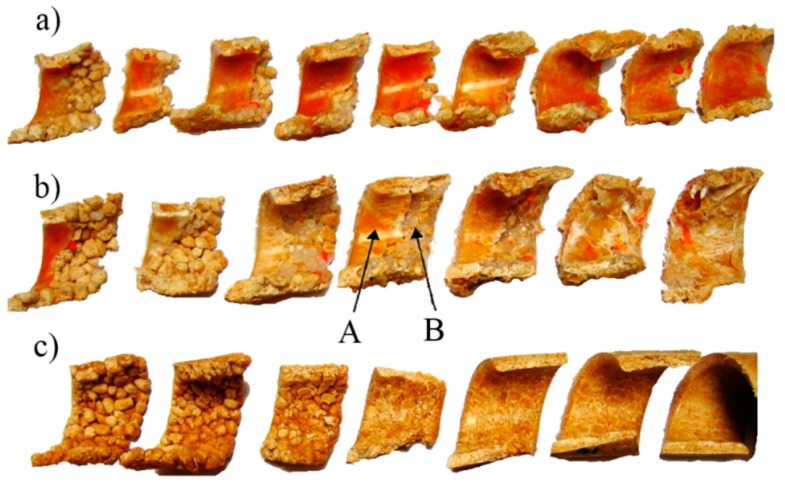
Melting of a wood-plastic composite of polypropylene PP and wood flour WF of a different composition in the single screw extrusion: (**a**) 25% WF, (**b**) 50% WF, (**c**) 75% WF, A—molten material, B—solid material [129] (with permission from Int. Polym. Process. 2015, 30, 113-120, by Wilczyński, K.; Nastaj, A., Lewandowski, A., Wilczyński, K.J., Buziak, K. © Carl Hanser Verlag GmbH & Co. KG, Muenchen).

**Figure 6:**
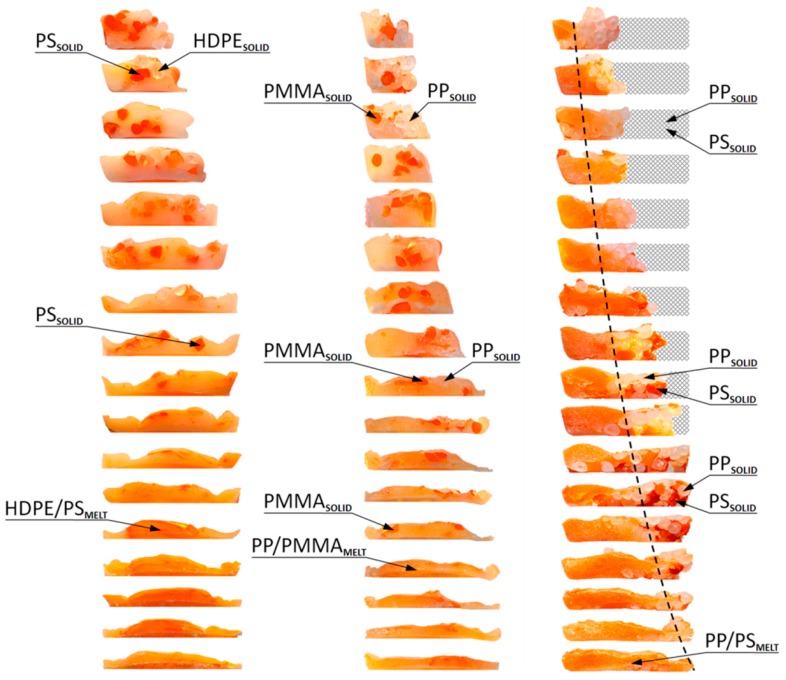
Melting of polyblends: (**a**) high density polyethylene/polystyrene blend (HDPE/PS)—starve fed extrusion, (**b**) polypropylene/polymethyl methacrylate blend (PP/PMMA)—starve fed extrusion, and (**c**) polypropylene/polystyrene blend (PP/PS)—flood fed extrusion [131].

**Figure 7:**
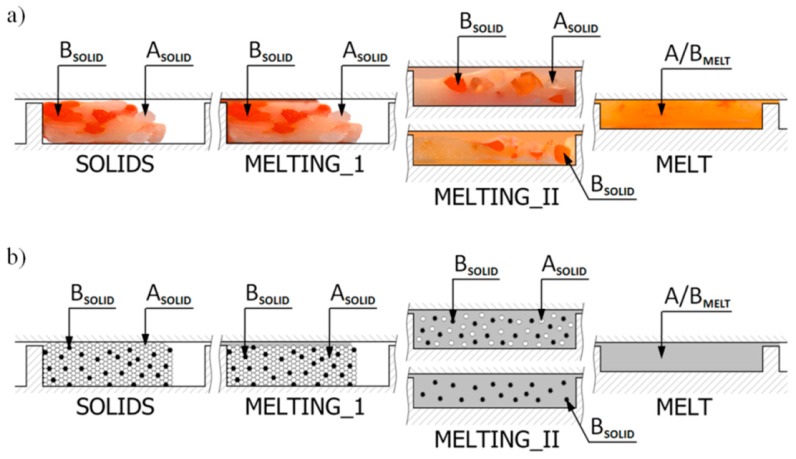
Melting of polymer blends in starve fed single-screw extrusion: (**a**) melting visualization, (**b**) melting model: A—major component of polyblen (HDPE), B—minor component of polyblend (PS), A/B—polyblend (HDPE/PS), MELTING_I—by heat conduction, MELTING_II—by energy dissipation [131].

Recently, Wilczyński et al. [129,130,131] observed different melting mechanisms in single screw extrusion, both flood-fed and starve fed, in the case of wood-polymer composites (Figure 5) and polymer blends (Figure 6 and Figure 7).

The methods of investigating and modeling the extrusion process were adapted to modeling injection molding. Experimental studies of melting in injection molding machines were performed first by Donovan et al. [132], who revealed that the screw recharge process is a transient plasticating extrusion process, which gradually approaches the equilibrium extrusion behavior as the screw rotates. If the screw rotation time is a high fraction of the total cycle time, the plasticating behavior is similar to the extrusion behavior. However, if the screw rotation time is a small fraction of the total cycle time, the plasticating behavior is significantly different. Donovan [133,134] also proposed a heuristic model for predictive simulations, which required experimental evaluation of an empirical parameter, specific to a particular material over the tested range of operating conditions. Lipshitz et al. [135] developed a theoretical model for melting, which was built upon the detailed physical mechanisms taking place in the reciprocating screw injection molding machine. This model permits the calculation of the solid bed profile as a function of time during the injection cycle. It consists of a dynamic extrusion melting model for the rotation period, a transient heat conduction model with a phase transition for the screw rest period, and a model for the drifting of the beginning of melting during the injection cycle.

**Figure 8:**
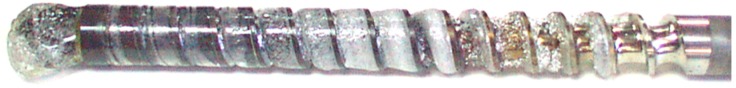
Melting mechanism for injection molding [141].

Later, the basic research in this field was performed first by Potente et al. [136,137], and then by Steller et al. [138,139] as well as by Covas et al. [140]. Recently, Wilczyński et al. [141] performed experimental studies on the melting mechanism of polymers in injection molding machines. It has been observed that melting in the injection molding machine occurs to some extent, according to the Tadmor mechanism, with clearly visible starvation (Figure 8).

The existing models of the injection molding process (plasticating unit) [136,137,138,139,140] differ from the extrusion models in that they involve the static and dynamic phases of melting (stationary and rotating screw) with an axial screw movement. However, it is assumed that the screw is fully filled with a material such as in the flood fed extrusion (Figure 2), which is inconsistent with Figure 8 where starvation is clearly seen in the starve fed extrusion (Figure 4).

### 2.3. Melt Conveying Section

The first analysis of flow in a single screw machine (screw pump) was performed by Rowell and Finlayson [142] for viscous oils who modeled the drag flow and pressure flow for an isothermal Newtonian fluid. This analysis was rediscovered by Carley et al. [143] and applied to the screw extrusion of polymers. It is not well known among the experts that Maillefer [144] developed nearly the same equations ahead of the publication of Carley et al. [143]. 

These basic studies analyzed one-dimensional flow through a rectangular channel of infinite width. Later, these models were improved by considering the transverse flow caused by the screw flights (Carley and Strub [145], Squires [146]), the effect of channel curvature (Booy [147], Squires [148]), and the effect of flight clearance (Mallouk and Mc Kelvey [149], Maddock [150]). 

In later analyses, the simplest non-Newtonian case was considered, which is a one-dimensional isothermal flow of the power-low fluid in a channel of infinite width, both analytically (Kroesser and Middleman [151], Middleman [152]) and numerically (Colwell and Nicholls [153]). Afterward, two-dimensional flow was considered (Griffith [154], Zamodits and Pearson [155]). These basic studies were summarized and expanded first by McKelvey [1] and then by Tadmor and Klein [2].

Since the pioneering and fundamental work of Tadmor and Klein [2], many researchers have attempted to improve the basic models by considering two-dimensional or three-dimensional flow, taking into account the non-Newtonian characteristics of the polymer melt and the actual screw geometry or using a better approach for the thermal analysis, while considering mechanical/thermal coupling (Fenner [156], Lindt et al. [157,158], Lawal and Kalyon [159], Spalding et al. [160], Syrjala [161,162], and Ilinca and Hetu [163]). Recently, Miethlinger et al. [164,165,166] proposed a heuristic method for two-dimensional or three-dimensional modeling of the flow of power-law fluids in metering sections of single screw extruders.

In addition to the primary flow field in the metering section of the single-screw extruder, for sharp flight screw root corners, Polychronopoulos and Vlachopoulos [167] determined a secondary flow in front of the root of the pushing flight and behind the root of the trailing flight, akin to what is known in fluid mechanics as Moffatt eddies. Due to extremely large times required for fluid particles to travel along the helical Moffatt eddy path lines, degradation is likely.

It should be noted in this case that, when modeling the flow in the melt conveying section, most authors assume the screw is stationary and the barrel rotates. Campbell and Spalding [9] take the position that the rotating barrel and rotating screw produces significantly different results in modeling. The detailed discussion of these two approaches has been performed by Rauwendaal [8].

Contrary to melt conveying in single screw extruders, the studies on melt conveying in twin-screw extruders were limited. The first experimental studies of flow in co-rotating twin-screw extrusion were performed by Erdmenger [168,169] who observed that material moved forward in the machine in a roughly helical eight-figure motion. The pumping mechanism of the co-rotating twin-screw extruder is a drag-induced flow much like that of the single-screw extruder. The geometry of the co-rotating intermeshing twin-screw configuration was studied in detail by Booy [170]. Newtonian flow models for fully filled elements were developed by Booy [171], Denson and Hwang [172], Szydłowski and White [173,174], and Tayeb et al. [175,176]. Later, non-Newtonian models were developed, e.g., by White et al. [177,178,179,180] and Potente et al. [181]. Todd [182] discussed the drag and pressure flows in twin-screw extruders. Recently, fully three-dimensional non-Newtonian FEM (Finite Element Method) computations were performed and the state-of-the-art tool was discussed by Ilinca and Hetu [163], Malik et al. [183], and Vergnes et al. [184] who compared the results of 3D simulations to the results issuing from the 1D Ludovic software. The 3D simulation method was found to be more accurate to describe flows in kneading discs, but the 1D model provided very satisfactory results for flows in screw elements. Several recent papers on modeling of co-rotating twin-screw extrusion may also be cited in this case [185,186,187,188,189,190,191].

Intermeshing counter-rotating twin-screw extruders are fundamentally different from single-screw machines, as well as from co-rotating twin-screw machines. They were first discussed by Kiesskalt [192], Montelius [193], and Schenkel [16] as positive displacement pumps whose throughput is controlled by screw geometry and screw speed. Doboczky [55,194] and Janssen et al. [117,195,196] developed flow pumping characteristics for these machines, and they gave primary attention to understanding the leakage flows between the screws, and between the screws and barrel. White and Adewale [197] developed a more general flow model considering the level of intermeshing in the machine. A numerical FEM simulation for an intermeshing counter-rotating twin-screw extruder was presented by Li and Manas-Zloczower [198], and by Kajiwara et al. [199]. However, no attention was given to screw pumping characteristics. Hong and White [200,201] presented a FAN analysis (Flow Analysis Network) of flow in this machine, and applied this method to non-Newtonian flow behavior. They have determined screw characteristic curves for various screw elements. This allowed us to model the flow for various modular screw designs and calculate pressure, fill factor, and temperature profiles. Schneider presented the historical development of the counter-rotating twin-screw extrusion [202]. Recently, Wilczyński and Lewandowski [203] performed a fully three-dimensional non-Newtonian FEM computation to design the screw pumping characteristics for counter-rotating extruders. An analysis included the flow in the C-chamber, and the leakage flows were identified over the calender gap, tetrahedron gap, flight gap, and side gap.

Currently, 3D FEM computations are available for single-screw extrusion and twin-screw extrusion, for which flow simplifications are minimized. These approaches accurately describe the velocity and temperature distributions and the pressure/flow rate relationships, but they require large computing resources and major calculation time. The POLYFLOW software package [204] can be used for simulating various aspects of extrusion including SSEs, TSEs, and die flows including viscoelastic effects.

**Figure 9:**
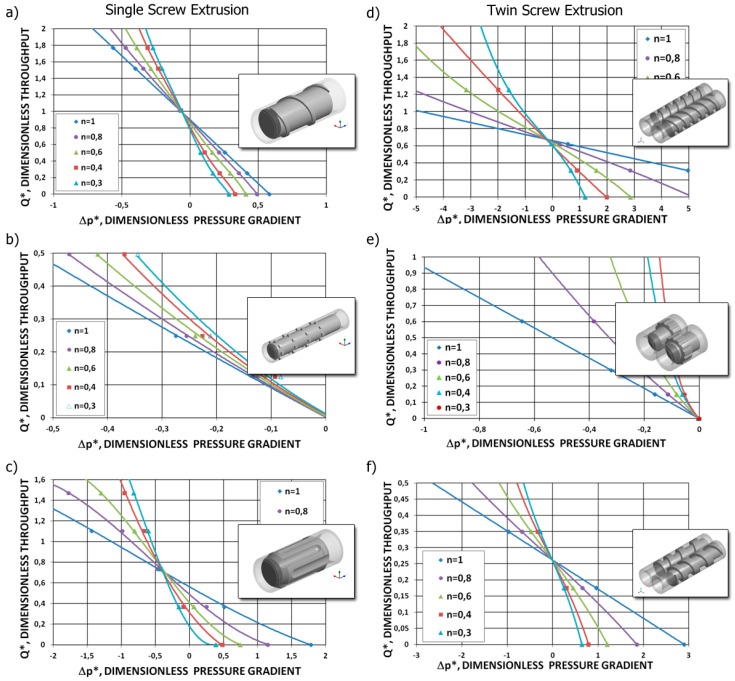
Screw pumping characteristics [128,206]: single-screw extrusion, (**a**) conventional screw, (**b**) mixing section, (**c**) Maddock section, counter-rotating twin-screw extrusion, (**d**) thick flighted section, (**e**) shearing section, and (**f**) thin flighted section.

For the major calculation time, these approaches cannot be used for global modeling of the extrusion process, which requires hundreds of computing iterations. In order to avoid the time-consuming computations during each iterative computing loop, the concept of screw pumping characteristics was developed, which are defined as the functional dependencies of the dimensionless flow rate and dimensionless pressure gradient [4]. These characteristics can be modeled by regression analysis and then implemented into the iterative computations by providing reasonable computation accuracy and computation time. Such characteristics were developed both for single-screw extrusion and twin-screw extrusion, e.g., by White and Potente [4], Rauwendaal [8], and by Wilczyński et al. [128,203,205,206], which are shown in Figure 9.

## 3. Computer Models of Extrusion

In the modeling of polymer processing, the models are generally deterministic, transport phenomena-based, either steady (continuous processes) or unsteady (cyclic processes), and of distributed parameters or locally lumped parameters. For engineering purposes, the lumped parameter models may be generally sufficient. The main goal of engineering designs is to predict the pressure and mean polymer melt temperature profiles along the machine for a given screw and die geometry as a function of the process operating conditions. In these models, the screw channel is usually divided into short axial segments (increments), where the input temperature and pressure parameters come from the calculation in the previous segment. In addition, the output parameters from the current segment are the input parameters for the next segment. Within each segment, the local parameters are assumed to be constant. 

The lumped parameter approach becomes particularly useful when dealing with plasticating processes, like extrusion and injection molding, where, in addition to the melt flow, we are faced with the solids’ transport and melting of the material.

With immense progress in the computational fluid dynamics, the current trends in modeling polymer processing apply very sophisticated numerical (e.g., finite element) methods. This includes both two-dimensional and three-dimensional computations of velocity, stress, pressure, and temperature fields with a variety of boundary conditions for shear-thinning and temperature-dependent, sometimes viscoelastic, fluids. In real extruders and injection molding machines, however, there is a lot of other problems that are still unsolved, and, at present, these methods are generally not applied for comprehensive (global) modeling of these screw processes.

The state-of-the-art for composite modeling of screw processes was presented in some fundamental books, e.g., by White and Potente [4], Rauwendaal [8], and Agassant et al. [11], as well as in some review papers, e.g., by Ariffin et al. [207], Wilczynski et al. [208], Teixeira et al. [209], and Malik et al. [183]. Single-screw extrusion, twin-screw extrusion, both co-rotating and counter-rotating, and injection molding were considered. The flood fed and starve fed operations were also discussed.

Tadmor and Klein [63] developed the first computer program EXTRUD for simulation of the extrusion process, which was described in Reference [2]. Afterward, Klein and Klein [210] presented the SPR (Scientific Process Research) extrusion simulation system. Next, several other computer programs for a single-screw extrusion were developed, e.g., Agur and Vlachopoulos [211] developed the NEXTRUCAD program, Potente et al. [212,213] built the REX program, Sebastian and Rakos [214] presented the PASS system (Polymer Analysis Simulation System), and Wilczyński [215,216] developed the SSEM program (Single Screw Extrusion Model). Other computer models were developed by Fukase and Kunio [217], Zavadsky and Karnis [218], Vincelette et al. [219], and Amellal and Lafleur [220]. Recently, Wilczyński et al. [221] developed the computer program for simulating the single-screw extrusion of wood polymer composites. 

Research on the co-rotating twin-screw extrusion was initiated by White and his co-workers. On the basis of the melt flow studies [173,174] and the polymer melting studies [52,113], the computer model of co-rotating twin-screw extrusion Akro-Co-Twin [222,223,224] was developed. Independent studies performed by Potente [53,112,181] led to the development of the SIGMA program [225,226]. The research carried out by Vergnes et al. [114,115,176] led to the development of the LUDOVIC program [227]. Canedo [228] built TXSTM program, and Teixeira et al. [209] developed the global software for co-rotating extruders.

Research on the counter-rotating twin-screw extrusion was also initiated by White and his co-workers. Based on the melt flow studies [200,201] and the polymer melting studies [56,120] (Figure 3), the first computer model of counter-rotating twin-screw extrusion Akro-Counter-Twin was developed [229,230]. These studies were continued by Wilczyński et al. [231,232] who developed the TSEM program (Twin Screw Extrusion Model). 

Research on the starve fed single screw extrusion was much more limited. Wilczyński et al. based on the polymer melting studies [59,127] developed the first, and, up to now, the only available computer model of this process SSEM-Starve [128]. This model was later extended to non-conventional screw configurations [205,206], and to the extrusion of polymer blends [233,234]. Recently, the global model GSEM (Global Screw Extrusion Model) was developed, which allows the modeling of single screw extrusion both in the flood fed and starve fed mode [205].

When modeling polymer extrusion, it is generally assumed that there is no slippage at the fluid/solid interface, and flowing materials in the screw extruders and dies adhere to the wall. However, there are several materials like filled polymers (e.g., wood polymer composites), elastomers, polymers like poly(vinyl chloride) and high-density polyethylene, and polymer suspensions, which exhibit wall slippage under certain conditions.

The phenomenon of wall slippage was studied first by Mooney [235]. Afterward, several studies were performed to answer how best to consider wall slippage when designing extruders. An extensive review on this subject was presented by Potente et al. [236]. Worth and Parnaby [237] presented an analysis of the effects of the wall slip on the extrusion throughput and power consumption for a one-dimensional isothermal Newtonian flow, and reported that power consumption in the extruder is reduced as a result of wall slippage. Meijer and Verbraak [238] performed two-dimensional Newtonian isothermal analysis, and showed an influence of slip on the velocity profiles and pumping characteristics of the extruder. Lawal and Kalyon [239,240] developed an analytical model describing single-screw extrusion of viscoplastic fluids with different slip coefficients at screw and barrel. Kalyon et al. [241] as well as Malik et al. [183] studied numerically co-rotating twin-screw extrusion with wall slippage at the barrel and screws. Potente and his co-workers, e.g., [236,242,243,244,245,246] performed very extensive studies on modeling single-screw extrusion with slip effects, both analytically and numerically, by calculating the pressure/throughput and drive power behavior, as well as the melt temperature development in single-screw extrusion of wall-slipping polymers. Several studies were performed on modeling the flow of wall slipping polymers in the dies, e.g., by Ferras et al. [247], Hatzikiriakos and Mitsoulis [248], and Gupta [249].

**Figure 10:**
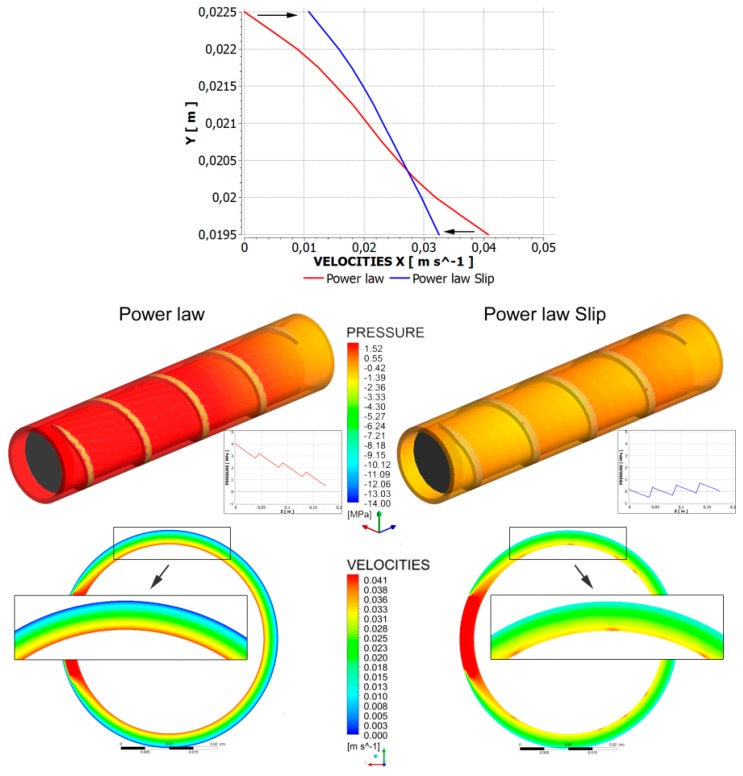
Screw flow simulations: pressure/velocity distributions for the power law model at slip/no slip conditions [251].

**Figure 11:**
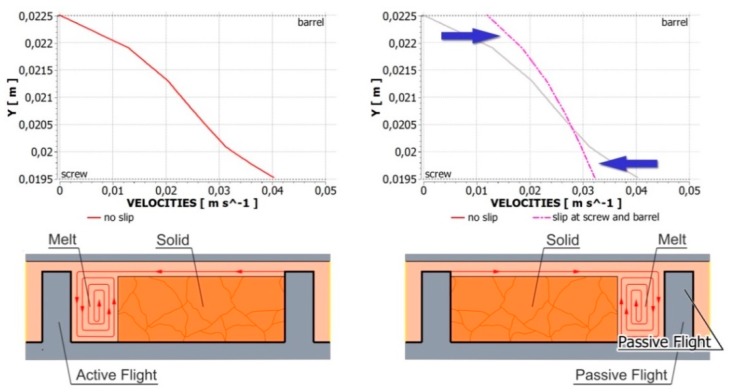
Slip effects and melting: (**a**) velocity distribution without a slip, and with slipping, and (**b**) possible melting mechanisms.

Recently, Lewandowski and Wilczyński [250,251] performed an extensive fully three-dimensional non-Newtonian FEM study on the polymer melt flow with slip effects in the single-screw extruder to design the screw/die pumping characteristics, which may be implemented into the composite model of the process. An analysis was performed for the flow of polymers with slip effects both in the screw (on the screw and barrel surfaces) and in the die. An example of simulation is depicted in Figure 10, which shows slipping at the screw/barrel surfaces. A possible melting mechanism changing, as reported in the literature [65,66], is depicted in Figure 11. The molten material may accumulate at the passive flight of the screw, which is not consistent with the Tadmor mechanism of melting.

Currently, it is established that extrusion of wall-slipping polymers results in the reduction of the die pressure, and the screw characteristics changing, which affects the operating point of the extruder, and results in the global modeling of extrusion of wall-slipping polymers. This requires developing models both for the screw (plasticating unit) and for the extrusion die.

When modeling polymer extrusion, it is also generally assumed that flowing materials in the screw extruders and dies have no yield stress. However, it is well known that many materials have a yield stress, e.g., filled polymers, composites, and blood, paints, cosmetics, and foodstuffs such as margarine, mayonnaise, butter, and ketchup. These materials were first described by Bingham [252], and, later, a number of works related to viscoplastics were reviewed by Bird et al. [253] and Mitsoulis [254].

Compared to fundamental studies on the viscoplastic flows, much less research was devoted to the viscoplastic flows in the extrusion process. Laval and Kalyon [239,240] first developed analytical models of the single-screw extrusion of viscoplastic fluids described by the Herschel-Bulkley model. Later, Kalyon et al. [241] presented a combined experimental and finite element study of the flow and heat transfer in twin-screw extrusion of concentrated suspensions using the Herschel-Bulkley model. 

**Figure 12:**
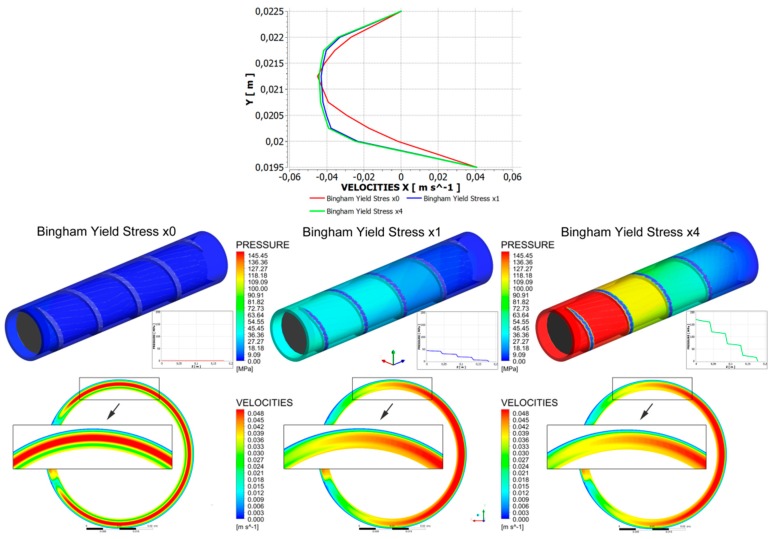
Screw flow simulations: pressure/velocity distributions for Bingham model [251].

Recently, Lewandowski and Wilczyński [251,255] performed an extensive fully three-dimensional non-Newtonian FEM modeling study on the viscoplastic flows in the single-screw extruder to design the screw/die pumping characteristics, which may be implemented into the composite model of the process. An analysis was performed for the flow with yield stress effects both in the screw and in the die. An example of simulation is depicted in Figure 12 where the flat velocity profile in the central part of the flow may be seen for some yield stresses.

## 4. Global Modeling of the Extrusion Process

Extrusion is a continuous process of co-operation of the extruder (screw) and the die. Physical phenomena occurring in the extruder determine the flow in the extrusion die and vice versa. The flow in the die determines the phenomena occurring in the extruder. Any change in the processing conditions in the extruder causes a change in the processing conditions in the die and vice versa. Modeling of the extrusion process cannot be limited to the modeling of flow in the extruder and must include the extrusion die.

The term global modeling means modeling the interacting phenomena occurring in the extruder and the die, which entails modeling the extruder/die system (or screw/die system).

**Figure 13:**
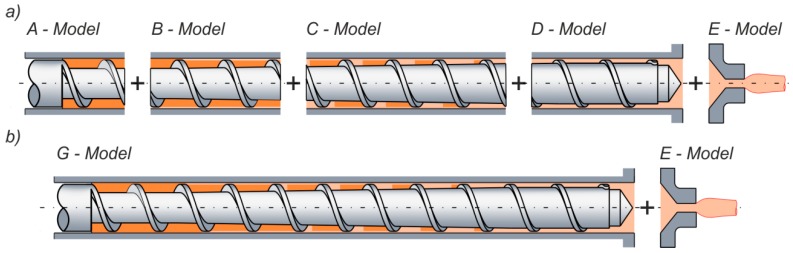
Modeling concepts: (**a**) classical modeling: A—solid conveying model, B—pre-melting model, C—melting model, D—melt conveying model, E—die flow model, (**b**) continuum modeling: G—continuous model.

The classical global modeling of the extrusion process is based on the separate models for each section of the screw, i.e., solid transport section, melting and pre-melting sections, and melt flow section, and the global model consists of these elementary models (Figure 13). In this case, the computations are performed step-by-step in the elementary segments of the screw, and the output process parameters from the current segment are the input parameters for the next one.

**Figure 14:**
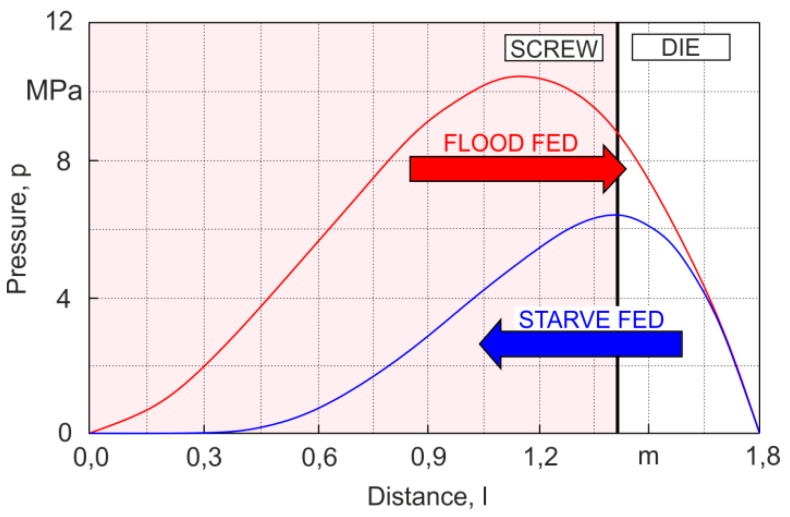
A forward scheme of computations for flood fed extrusion, and a backward scheme of computations.

Global modeling requires the use of a computation algorithm appropriate for a given type of extrusion. For classical (flood fed) extrusion, the forward scheme of computation is suitable. In this case, the flow rate of the material is not known, and is the result of the screw/die co-operation and must be determined in multiple iterative computations. For extrusion with starvation, the backward scheme of computation is applied, i.e., the inverse computation algorithm since the pressure profile is not continuous in this scenario, and there is no continuous flow rate/pressure relation. In this case, the flow rate is established and equal to the flow rate of the material metered by the dosing device (Figure 14).

**Figure 15:**
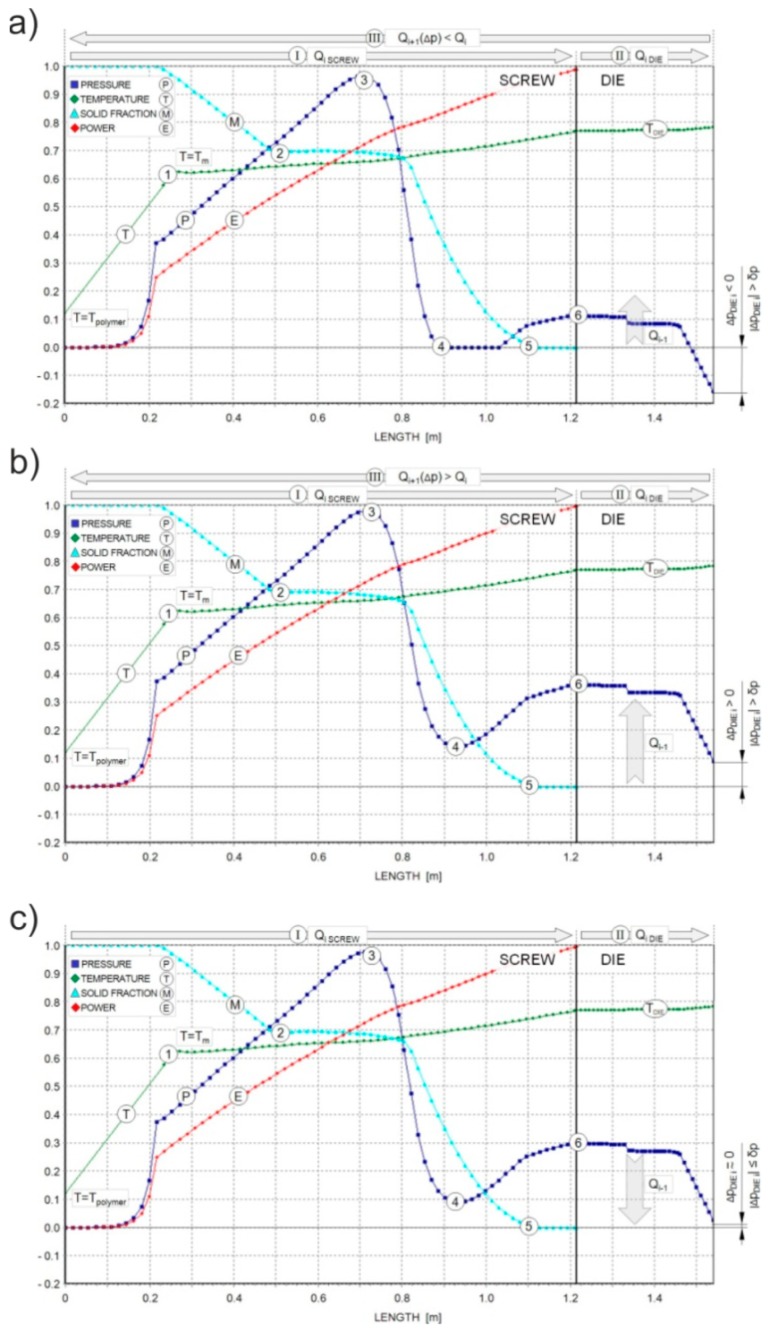
Scheme of computation for flood fed single screw extrusion: (**a**) Δ*p*_die_ < 0, (**b**) Δ*p*_die_ > 0, (**c**) ׀Δ*p*_die_׀ < δ_p_, 1—start of melting, 2—start of compression section, 3—pressure max, 4—end of pressure drop, 5—end of melting, 6—pressure at screw exit (die inlet), *Q*—flow rate, *Q_i_*_+1_—next iteration flow rate, *p*—pressure, Δ*p*_die_—die pressure at die exit, δ_p_—accuarcy of pressure computation, *T*—temperature, *T*_m_—melting point, *T*_die_—die melt temperature, *M*—solid fraction (melting), *E*—power consumption.

Simulation schemes for flood fed single-screw extrusion are relatively well known [210,211,212,213,214,215,216]. In this case, the modeling proceeds from the hopper to the die, according to the forward scheme of calculations (Figure 14), and the extrusion operating point is searched, which defines the extrusion flow rate and die pressure. The flow rate is not known, and results from the extruder/die co-operation. Computations start for some presumed flow rate, e.g., equal to the drag flow rate, and solid conveying, melting, melt conveying, and die flow are simulated. The calculated pressure at the die exit is compared to the atmospheric pressure. The computation is achieved when both pressures are equal. Otherwise, the presumed flow rate is modified and computations are iteratively repeated until the convergence is reached. A scheme of such computations is depicted in Figure 15. The arrows indicate the direction of computations, which are forward computations in the screw (I) and die (II) sections, and a backward step (III) to repeat computations for the new flow rate. 

In the case depicted in Figure 15, the computations are repeated for a modified presumed flow rate, and, after hundreds of iterations, the convergence is reached (Figure 15c).

**Figure 16:**
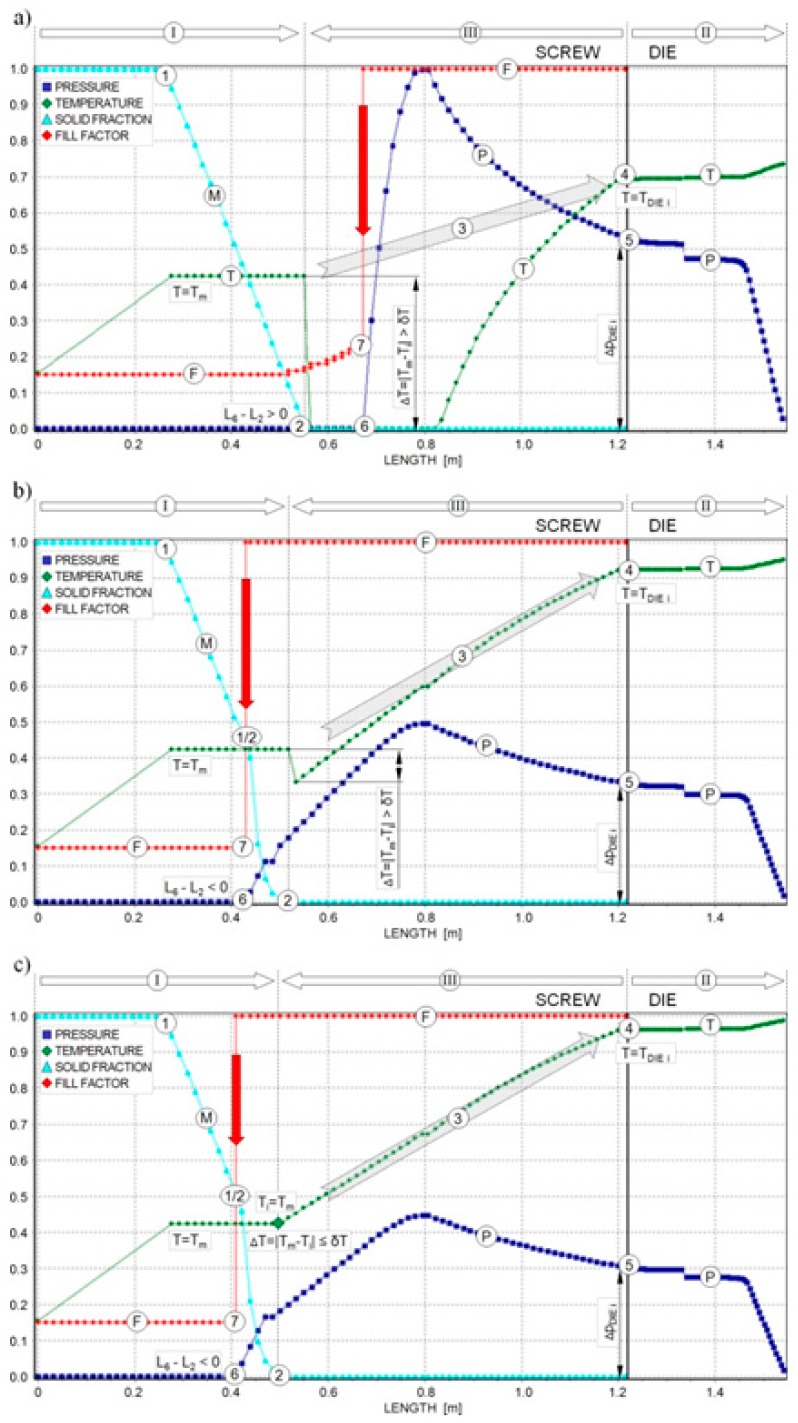
Computation scheme: (**a**) one-stage melting mechanism, computation discrepancy, (**b**) two-stage melting mechanism, computation discrepancy, (**c**) computation convergency: (I), forward computations in the melting section, (II) forward computations in the die section, (III), backward computations in the melt conveying section, (*M*), melting (*SF*), (*F*), filling (fill factor), (*T*), temperature, (*P*), pressure, (1), start of melting, (2) end of melting, (3) transfer of computations to the die, (4) start of die melt temperature computation, (5) start of die pressure computation, (6) zero pressure location, (7) beginning of filling computation (partly filled region starts), ΔP_DIEi_, die pressure, *T*_DIEi_, presumed melt temperature, *T*_m_, melting point, *i*, number of iterations, Δ*T* = |*T*_m_ − *T*_i_|, convergency checking, and δ*T*, computation accuracy [206].

Simulation schemes for starve fed single-screw extrusion are much less known [128,205,206]. In this case, the modeling requires an inverse approach. The flow rate is known, and is equal to the feeding rate. However, since the screw is not completely filled with the polymer, the computations cannot be processed from the hopper to the die. In this case, the die pressure is computed first for a presumed polymer melt temperature. Then, the pressure gradient along the screw is computed using the screw pumping characteristics. When the pressure falls to zero, the starvation begins and the screw filling is computed. The calculated temperature at the end of melting is compared to the melting point, and the computation is achieved when both temperatures are equal. Otherwise, the presumed melt temperature is modified and computations are iteratively repeated until the convergence is reached. A scheme of such computations is depicted in Figure 16. The arrows indicate the direction of calculations, such as forward calculations in the melting (I) and die (II) sections, and backward calculations in the melt section (III).

In the case depicted in Figure 16, the computations are repeated for a modified presumed exit temperature, and the second stage of melting appears (Figure 16b). Thus, the computation scheme gets much more complicated since the location of the transition partially/fully filled screw has to be determined. At this location, the second stage of melting starts, and the second melting mechanism is included into the computations. After hundreds of iterations, the convergence is reached (Figure 16c).

Using this inverse computation approach, the authors developed the composite models for closely intermeshing counter-rotating twin-screw extruders [229,231,232]. This was also applied by other researchers for co-rotating twin-screw extruders [222,226,227]. However, those composite models using one-stage melting models were much simpler in execution. Moreover, the location of the melting regions was not computed but specified previously in those cases.

## 5. Future Concepts 

In the extrusion process, both single-screw extrusion and twin-screw extrusion (co-rotating and counter-rotating), flood fed or starve fed, the modeling of polymer melting consists of performing an experiment to get to know the melting mechanism, then proposing the physical model of this mechanism, and, lastly, developing the mathematical model. Thus, these models are not overall models, and are limited to the specific extrusion method.

**Figure 17:**
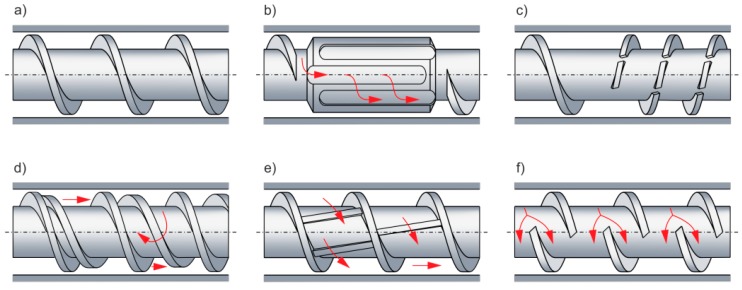
Conventional and non-conventional screw configurations: (**a**) conventional section, (**b**) Maddock section, (**c**) mixing section, (**d**) Maillefer section, (**e**) Barr section, and (**f**) Rheotoc section.

These models are also not general in nature because of the material being processed. For example, melting of polymers that exhibit slippage during the flow does not follow the Tadmor model because the molten material does not accumulate at the active flight of the screw, but flows toward the passive flight. These models are also not general in view of the operating and geometrical parameters of the process. For example, the melting mechanism may vary substantially depending on the screw geometry (Figure 17) as well as on the screw speed.

In conclusion, the currently available extrusion models are not general in nature due to limitations of the extrusion type, specific material, and specific operating and geometrical conditions of the process. Therefore, the question arises whether it is possible to solve the problem of global modeling of the extrusion process without referring to the specific solid/melt flow mechanism, which is determined by the extrusion method, the material type, and the process conditions. 

So far, there is no global model of the extrusion process, which would not be limited in this range. Therefore, the concept may be proposed to solve this problem on the basis of fluid mechanics using CFD (Computational Fluids Dynamics) computation procedures. 

**Figure 18:**
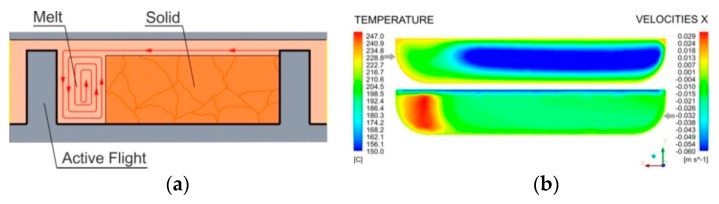
Example of modeling: (**a**) geometrical model of the melting mechanism, and (**b**) temperature and velocity distribution in the cross-section of the screw channel.

Instead of the specific solid/melt flow mechanism assumed, the polymer flow in the extrusion process can be simulated by solving the conservation equations of mass, motion, and energy along with a polymer constitutive equation, which was proposed by some researchers for the melting section [100,101,102,103,104,105]. An example of such computations is depicted in Figure 18.

This novel approach based on solving the conservation equations without an assumed polymer flow mechanism seems to be very promising for the future work on modeling the extrusion process, even though it requires very large computational capabilities. 

**Figure 19:**
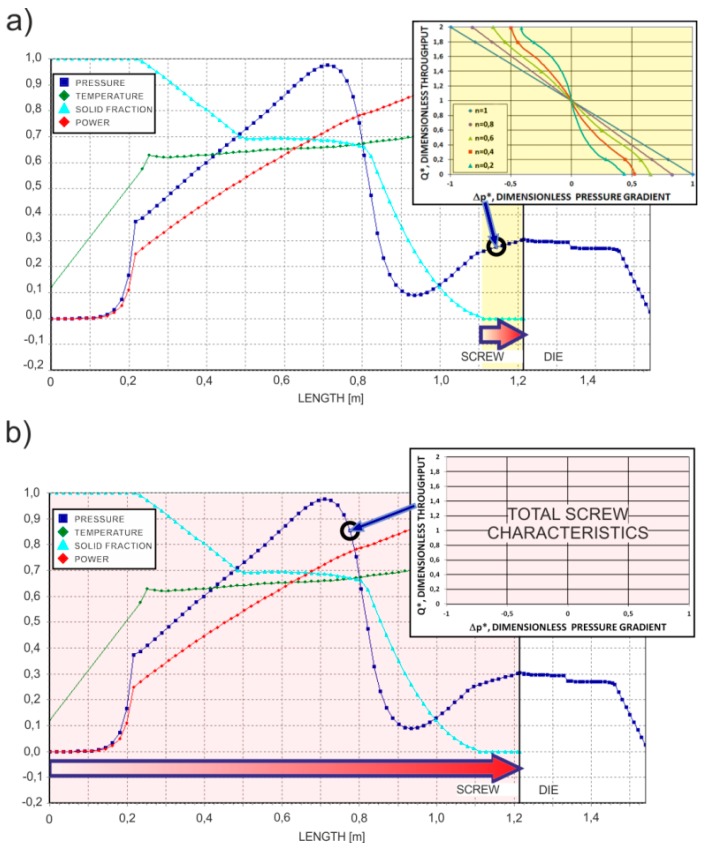
Various concepts of implementation of screw characteristics into the global model of the extrusion process: (**a**) screw pumping characteristics implemented into the melt region, (**b**) total (continuous) screw characteristics implemented into the entire area of the screw. Example of modeling.

The global modeling of the extrusion process, however, requires iterative computations to define the process operating point, and the question arises regarding how to implement the continuum computations into the global model of the process. This might be solved by developing a novel concept of the total (continuous) screw characteristics, which is schematically depicted in Figure 19, similarly to the screw pumping characteristics valid for the melt flow region [128,203,205,206], which are shown in Figure 9.

The most promising approach would be the coupled DEM/CFD modeling. Very recently, a coupling between EDEM, a DEM software, and OpenFOAM, which is an open-sourced computational fluid dynamics CFD software, has been developed and is now under testing. The initial version of this coupling allows for momentum transfer between EDEM and OpenFOAM, with a heat transfer capability to be implemented. The coupling overcomes one of the common limitations with DEM/CFD coupled simulations, namely that particles must be smaller in volume than the mesh cells they occupy, which allows for a wide range of application types that were previously not possible.

## 6. Conclusions

An issue of global modeling of polymer extrusion was reviewed and discussed, which includes modeling for solid conveying, melting, and melt flow, as well as co-operation of the screw/die system.

It was observed that the basis of computer extrusion models are still the early models of solid conveying and polymer melting, and no significant progress has been made in recent years. Extrusion modeling using a discrete element method (DEM) for solid conveying, and computational fluid dynamics (CFD) for polymer melting may be promising in this respect.

It was also observed that the extrusion models are generally limited to neat polymers. When modeling the polymer extrusion, it is assumed that there is no slippage at the fluid/solid interface, and flowing materials in the screw extruders and dies adhere to the wall. It is also assumed that flowing materials have no yield stress. However, there are many materials like filled polymers (e.g., wood polymer composites) that have a yield stress and exhibit wall slippage under certain conditions.

Limitations of the traditional approach to modeling based on an assumed polymer solid/melt flow mechanism were presented, and the novel continuous concept of global modeling based on solving the conservation equations without an assumed polymer flow mechanism was proposed. A concept of the total (continuous) screw characteristics was discussed to solve the problem of implementing the continuum computations into the global model of the process. Promising progress in coupled DEM/CFD modeling was indicated.
